# Improving the cost-effectiveness of cardiovascular disease prevention in Australia: a modelling study

**DOI:** 10.1186/1471-2458-12-398

**Published:** 2012-06-01

**Authors:** Linda J Cobiac, Anne Magnus, Jan J Barendregt, Rob Carter, Theo Vos

**Affiliations:** 1 School of Population Health, University of Queensland, Herston, 4029, Australia; 2 Deakin Health Economics, Strategic Research Centre – Population Health, Deakin University, 221 Burwood Highway, Burwood, Victoria, 3125, Australia

## Abstract

**Background:**

Cardiovascular disease is the leading cause of death worldwide. Like many countries, Australia is currently changing its guidelines for cardiovascular disease prevention from drug treatment for everyone with ‘high blood pressure’ or ‘high cholesterol’, to prevention based on a patient’s absolute risk. In this research, we model cost-effectiveness of cardiovascular disease prevention with blood pressure and lipid drugs in Australia under three different scenarios: (1) the true current practice in Australia; (2) prevention as intended under the current guidelines; and (3) prevention according to proposed absolute risk levels. We consider the implications of changing to absolute risk-based cardiovascular disease prevention, for the health of the Australian people and for Government health sector expenditure over the long term.

**Methods:**

We evaluate cost-effectiveness of statins, diuretics, ACE inhibitors, calcium channel blockers and beta-blockers, for Australian men and women, aged 35 to 84 years, who have never experienced a heart disease or stroke event. Epidemiological changes and health care costs are simulated by age and sex in a discrete time Markov model, to determine total impacts on population health and health sector costs over the lifetime, from which we derive cost-effectiveness ratios in 2008 Australian dollars per quality-adjusted life year.

**Results:**

Cardiovascular disease prevention based on absolute risk is more cost-effective than prevention under the current guidelines based on single risk factor thresholds, and is more cost-effective than the current practice, which does not follow current clinical guidelines. Recommending blood pressure-lowering drugs to everyone with at least 5% absolute risk and statin drugs to everyone with at least 10% absolute risk, can achieve current levels of population health, while saving $5.4 billion for the Australian Government over the lifetime of the population. But savings could be as high as $7.1 billion if Australia could match the cheaper price of statin drugs in New Zealand.

**Conclusions:**

Changing to absolute risk-based cardiovascular disease prevention is highly recommended for reducing health sector spending, but the Australian Government must also consider measures to reduce the cost of statin drugs, over and above the legislated price cuts of November 2010.

## Background

Cardiovascular disease is a leading cause of death and ill health in Australia [1]. Despite many decades in decline, with less smoking and more successful treatment, cardiovascular disease remains the number one cost to the Australian health sector [2], and its prevention has been declared a national priority [3,4].

Pharmaceuticals can be used to prevent ischaemic heart disease and stroke events by treating unhealthy blood pressure and lipid levels [5,6]. Around 10% to 20% of Australians without cardiovascular disease already report taking these preventive therapies, with at least half taking more than one drug [7,8]. However, some of these drugs, such as statins, are very expensive, and with rapidly increasing costs of the Pharmaceutical Benefits Scheme (PBS), it is imperative that we are identifying and providing drug treatment to those most at risk of going on to develop cardiovascular disease.

Australian guidelines for identifying those at risk are currently based on a confusing mix of rules and prescribing criteria for defining risk factor thresholds for ‘high blood pressure’ or ‘high cholesterol’ [9-12].

## A synthesis of current guidelines and prescribing criteria for prevention of cardiovascular disease in Australia

Criteria for treatment of blood pressure:

• Blood pressure > 140/90 mmHg

• Blood pressure > 130/80 mmHg and diabetes

Criteria for treatment of lipids:

• Diabetes and age > 60 years

• Diabetes and total cholesterol > 5.5 mmol/L

• Total cholesterol > 6.5 mmol/L and HDL cholesterol < 1 mmol/L

• Total cholesterol > 6.5 mmol/L and hypertension

• Total cholesterol > 5.5 mmol/L and HDL cholesterol < 1 mmol/L and hypertension

• Total cholesterol > 7.5 mmol/L or triglycerides > 4 mmol/L for men aged 35-75 years or post-menopausal women < 75 years

• Total cholesterol > 9 mmol/L or triglycerides > 8 mmol/L

However, in other countries such as New Zealand and the United Kingdom, recommended practice has progressed to screening and treatment based on a patient’s *absolute* risk [13], which takes additional risk factors, such as age, sex, smoking and diabetes, into account, alongside blood pressure and lipid levels. This is a more effective and cost-effective approach to cardiovascular disease prevention [14], and similar changes have been proposed for guidelines in Australia [15].

In this research, we model cost-effectiveness of cardiovascular disease prevention with blood pressure and lipid drugs in Australia under three different scenarios: (1) the actual current practice in Australia (based on self-reported use of blood pressure drugs, lipid drugs or both, in a national survey); (2) prevention as intended under the current guidelines (based on applying current risk factor threshold rules to survey participants’ measures of blood pressure and cholesterol); and (3) prevention according to proposed absolute risk levels (based on applying an Australian absolute risk prediction equation to survey participants’ age, sex, blood pressure levels, cholesterol levels, smoking status and diabetes status) . We consider the implications of changing to absolute risk-based cardiovascular disease prevention, for the health of the Australian people and for Government health sector expenditure over the long term.

## Methods

### Screening

We model cost-effectiveness of cardiovascular disease prevention in the 2008 Australian population aged 35 years and older [16]. The proportion of Australians currently taking blood pressure and lipid drugs (‘current practice’) is determined from self-reported use of cardiovascular disease drugs in those who have never experienced an ischaemic heart disease or stroke event in the 1999–2000 AusDiab dataset [7].

Since primary prevention screening occurs primarily in general practice in Australia, we determine the number of Australians who would be screened for cardiovascular disease risk from rates of general practice attendance in BEACH data [17] and estimates of general practitioner (GP) participation in risk assessment based on participation in Practice Incentive Programs. Eligibility for preventive drugs under the current mix of single risk factor-based guidelines and prescribing criteria is determined by the criteria defined in the synthesis of current guidelines and prescribing criteria for prevention of cardiovascular disease in Australia. Eligibility according to absolute risk is derived using the Framingham risk prediction equation [18]. Guidelines around measuring absolute risk in Australia [15] and New Zealand [19] are based on the 1991 version of the Framingham equation, which predicts the probability of a fatal or non-fatal ischaemic heart disease or stroke event in the next five years (in contrast to the ten-year prediction equation used in UK and European guidelines [13]). It is not possible to translate directly between the five and ten year predictions, because the risk of an event will increase with age, but as a rough rule-of-thumb we can say that a 5% five-year risk is approximately equivalent to a 10% ten-year risk and a 10% five-year risk is approximately equivalent to a 20% ten-year risk.

The Framingham risk is determined using the AusDiab data set for 1999–2000 [7]. Probability of an event in the next five years is derived from data on the age, sex, smoking status, total cholesterol level, high density lipoprotein cholesterol level and diabetes status of AusDiab participants who report never having experienced an ischaemic heart disease or stroke event. For participants reporting current use of blood pressure or lipid drugs, we assume a mean blood pressure-lowering effect of 9.1 mmHg (systolic) and 5.5 mmHg (diastolic), and a mean statin therapy effect of 17.1% reduction in total cholesterol, 25.6% reduction in low density lipoprotein cholesterol, 9.3% reduction in triglyceride and 3.3% increase in high density lipoprotein [5,6]. The Framingham risk prediction is then calibrated for the Australian population from the Framingham risk distribution in the AusDiab population who do not have IHD or stroke, and known Australian incidence of IHD and stroke [1]. We evaluate cost-effectiveness of cardiovascular disease prevention for three levels of risk, defined by the probability of a cardiovascular disease event in the next five years: ≥15% risk, ≥10% risk and ≥5% risk.

### Treatment

We evaluate cost-effectiveness for primary prevention with a combination of statin and blood pressure-lowering drugs (diuretics, ACE inhibitors, calcium channel blockers and beta-blockers). Current combinations of blood-pressure lowering drugs are derived from current usage patterns in the Avoid Stroke as Soon as Possible (ASAP) study [8]. For primary prevention according to the existing guidelines and absolute risk, we assume that blood pressure-lowering agents are prescribed in the most cost-effective order, with prescription of one, two or (at most) three blood pressure-lowering drugs based on the ASAP study data.

Reductions in risk of ischaemic heart disease and stroke associated with drug use are based on meta-analyses of primary prevention trials [5,6], with the effect of multiple drugs determined multiplicatively [20]. We use measures of statin drug efficacy for men and women combined, but also evaluate results with different measures of drug efficacy for men and women, based on the meta-analyses of trials that reported results by gender.

There is limited data on long-term adherence to blood pressure and statin drugs in primary prevention. We assume that 40% of patients will discontinue treatment at 12 months based on Australian data on discontinuation of statins [21] and blood pressure-lowering drugs [22]. Remaining patients are assumed to adhere to treatment long term, with twice-yearly visits to their general practitioner (GP). We do not include any additional costs (or effects) of intervention to improve or maintain adherence, such as patient education programs and reminders.

The unit costs of treatment are derived from Pharmaceutical Benefits Scheme (PBS) [23] data on scripts/services and benefits paid (taking the mix of ‘general’, ‘concession’, ‘safety net’ and ‘non-safety-net’ patients into account) and Medicare Benefits Schedule (MBS) costs for associated general practitioner visits and blood tests (Table 1 and Table 2). Where more than one brand or dose of drug is available, an average annual cost is determined from the 2008 PBS mix of scripts provided and the equivalent standard dose of the blood pressure-lowering [24] and statin drugs [25]. For comparison, we also consider results when statin drugs are costed at the cheaper price of statin drugs in nearby New Zealand.

**Table 1:** Costs of GP visits and blood tests for lipid and blood pressure-lowering therapy

	**Unit price (2008A$)**		**Number of units**		**Sources and assumptions**
	**Government**	**Patient**	**Year 1**	**Year 2+**	
Medical costs with lipid treatment*					
Long GP visit	$54.19	$9.56	1	−	MBS cost of Level C consultation [33]
Short GP visit	$28.52	$5.03	−	2	MBS cost Level B consultation [33]
Blood test – lipids	$15.13	$2.67	1	2	MBS cost for up to 6 test items (MBS Item 66512) [33]
Medical costs with blood pressure treatment*					
Long GP visit	$54.19	$9.56	1	−	MBS cost of Level C consultation [33]
Short GP visit	$28.52	$5.03	2	2	MBS cost Level B consultation [33]
Blood test – urea, electrolytes	$15.13	$2.67	3	2	MBS cost for up to 6 test items (MBS Item 66512) [33]
Medical costs with lipid and blood pressure treatment**					
Long GP visit	$54.19	$9.56	1	−	MBS cost of Level C consultation [33]
Short GP visit	$28.52	$5.03	2	2	MBS cost Level B consultation [33]
Blood test – lipids, urea, electrolytes	$15.13	$2.67	4	2	MBS cost for up to 6 test items (MBS Item 66512) [33]

* Patients eligible according to single risk factor thresholds.

** Patients eligible according to single risk factor thresholds or eligible according to absolute risk.

**Table 2:** Costs of lipid and blood pressure-lowering pharmaceuticals

	**Unit price (2008A$)**		**Number of units**		**Sources and assumptions**
	**Government**	**Patient**	**Year 1**	**Year 2+**	
**Pharmaceutical costs**					
Annual cost of low-dose diuretic therapy	$52.03	$18.79	1	1	Average annual PBS cost for the standard daily dose [24] of hydrochlorothiazide, chlorthalidone and indapamide, weighted by scripts provided in 2008 [23].
Annual cost of calcium channel blocker therapy	$163.66	$54.22	1	1	Average annual PBS cost for the standard daily dose [24] of verapimil, amlodopine (maleate), nifedipine, felodipine, amlodopine (besylate) and lercanidipine,weighted by scripts provided in 2008 [23].
Annual cost of ACE inhibitor therapy	$130.85	$81.21	1	1	Average annual PBS cost for the standard daily dose [24] ofcaptopril, fosinopril, enalopril, ramipril, quinapril, lisinopril, trandolopril and perindopril, weighted by scripts provided in 2008 [23].
Annual cost of beta-blocker therapy	$169.59	$47.09	1	1	Average annual PBS cost for the standard daily dose [25]of fluvastatin, simvastatin, atorvastatin, pravastatin and rosuvastatin, weighted by scripts provided in 2008 [23].
Annual cost of statin therapy	$508.64	$178.79	1	1	Average annual PBS cost for the standard daily dose [25] of fluvastatin, simvastatin, atorvastatin, pravastatin and rosuvastatin, weighted by scripts provided in 2008 [23].
Annual cost of statin therapy in New Zealand	$18.25	−	1	1	Average annual cost of simvastatin (40 mg/day) in New Zealand [38].
Annual cost of current practice lipid-lowering therapy	$559.68	$123.37	1	1	Average annual cost from actual PBS expenditure on lipid-lowering drugs in 2008 [23].
Annual cost of current practice blood pressure-lowering therapy	$169.59	$47.09	1	1	Average annual cost from actual PBS expenditure on bloodpressure-lowering drugs in 2008 [23]. Mix of diuretics, beta-blockers, calcium channel blockers and ACE inhibitors based on BEACH general practice data [32].

NB. All costs adjusted to 2008 Australian dollars using Australian health price deflators [39], consumer price index [40] and/or purchasing power parities [41] where relevant.

Costs are calculated in the model according to how many patients receive blood pressure drugs only, how many receive lipid drugs only and how many receive both blood pressure and lipid drugs. With single risk factor screening, some patients will receive blood pressure or lipid drugs only, while some will receive both. With absolute risk screening, however, all eligible patients receive a combination of both blood pressure and lipid drugs. We assume that all patients receiving treatment, no matter how they are screened, will have one long visit (at least 20 minutes) with their GP and one blood test (e.g. to determine lipid levels) in the first year of treatment. All patients are then assumed to have two short visits (up to 20 minutes) with their GP and two blood tests annually thereafter, for on-going monitoring and repeat drug prescription. Patients receiving blood pressure-lowering drugs (e.g. ACE inhibitors) also receive two additional blood tests and two short GP visits in the first year of treatment, for monitoring of urea and electrolyte levels.

### Cost-effectiveness modelling

In a discrete time Markov model, we simulate IHD and stroke events, by age and sex, over the lifetime of the Australian population without a history of cardiovascular disease in 2008, from age 35 years. The Markov model has four primary health states, with transition rates capturing probabilities of incidence and case fatality for fatal and non-fatal IHD and stroke events. Rates are derived from Australian hospital and mortality databases [26,27], the Perth MONICA study [28] and the NEMESIS [29] study. Trends are incorporated to capture underlying changes in IHD and stroke incidence and case fatality over time [30]. A full description of the model and data inputs is provided in the Additional File 1.

Prevention of cardiovascular disease under the three scenarios (actual current practice; prevention as intended under the current single risk factor-based guidelines; and prevention according to proposed absolute risk-based guidelines) is evaluated in comparison to no intervention for prevention of cardiovascular disease. Incidence of ischaemic heart disease and stroke under the comparator conditions of no intervention, are back-calculated using estimates of relative risks (from Table 3), the proportion of the population without cardiovascular disease who are taking preventive cardiovascular drugs as recorded in AusDiab [7] and BEACH general practice data [31,32].

**Table 3:** Model input parameters and their uncertainty distributions

**Parameter**	** Value** **Mean (SE or 95%CI)**	**Uncertainty distribution**	**Sources and assumptions**
RR of IHD with treatment			
-Statin -Diuretic -Calcium channel blocker -ACE inhibitor -Beta-blocker	0.70 (0.61 to 0.81) 0.86 (0.75 to 0.98) 0.85 (0.78 to 0.92) 0.83 (0.78 to 0.89) 0.78 (0.89 to 1.02)	Normal (lnRR)	Meta-analyses of primary prevention trials [5,6,47]
RR of stroke with treatment			
-Statin -Diuretic -Calcium channel blocker -ACE inhibitor -Beta-blocker	0.81 (0.71 to 0.93) 0.62 (0.53 to 0.72) 0.66 (0.58 to 0.75) 0.78 (0.66 to 0.92) 0.70 (0.83 to 0.99)	Normal (lnRR)	Meta-analyses of primary prevention trials [5,6,47]
RR of stroke in IHD			
-Men -Women	1.32 (0.20) 1.88 (0.30)	Normal (lnRR)	Busselton study [48,49]
RR of IHD in stroke			
-Men -Women	2.64 (0.07) 2.85 (0.04)	Normal (lnRR)	Busselton study [48,49]
IHD treatment cost			
-First year -Subsequent years	$12,921 $4,539	Uniform	Lim [43]. Uniform distribution assumed to vary by ±25% around mean.
Stroke treatment cost			
-First year -Subsequent years	$23,581 $3,201	Uniform	Lim [43]. Uniform distribution assumed to vary by ±25% around mean.
Proportion of population visiting a GP in one year			
35–44 yrs 45–54 yrs 55–64 yrs 65–74 yrs 75+ yrs	Men Women 73% 88% 81% 89% 81% 89% 93% 94% 99% 99%	–	BEACH data [17]
Proportion of GPs measuring absolute risk	65% (6.5%)	Beta	Practice Incentives Program data [50].
First year drug discontinuation rate	40% (8%)	Beta	Estimate from Australian survey data [21,22]. Standard error assumed to be 20% of point estimate.

NB. All costs adjusted to 2008 Australian dollars using Australian health price deflators [39]. RR – relative risk.

Using the Markov model, we simulate the difference between total years of life that would be lived by the population receiving preventive drug intervention under the three scenarios (taking uptake and adherence into account), and total years of life that would be lived if the same population did not receive any drugs for cardiovascular disease prevention. To capture the impact on morbidity, we adjust the years of life that are lived (in both intervention and comparator populations) using utility weights that capture the average quality of life experienced at each age and sex [34]. Time lived with heart disease or stroke is also weighted to reflect the associated health loss [35-37].

We simulate the impact of each scenario on costs of treating ischaemic heart disease and stroke over the lifetime of the population. Costs of treating ischaemic heart disease in the first year of illness are derived from a Victorian Government study [42] of hospital inpatient costs for ischaemic heart disease treatment and rehabilitation admissions, and from a study by Lim [43] of government and patient out-of-hospital costs for consultations, drugs and diagnostic procedures, and Medicare rebates for private-sector consultations and procedures. Costs of treating stroke in the first year after a stroke event and annual costs of treatment in subsequent years are derived from the NEMESIS study of stroke costs [44].

Total costs of intervention (to Government and patients), treatment costs that could be averted and overall population health impacts (measured in quality adjusted life years) are determined by simulating the population over time until everyone is dead or has reached 100 years of age. All future costs and health outcomes are discounted back to the baseline year at a rate of 3% [45]. We take the difference in cost of intervention to Government, between each scenario and current practice, to reflect the potential cost-savings in blood pressure and statin drug prescribing.

We derive 95% uncertainty intervals for all cost and health outcome measures from uncertainty around input parameters (Table 3) by multivariate probabilistic sensitivity analysis [46] using the Excel Add-In program @Risk (Palisade, Version 4.5). Finally, cost-effectiveness ratios are evaluated in Australian dollars per quality adjusted life year (QALY) for the year 2008, and compared with a value-for-money threshold of $50,000/QALY [51].

## Results

**Figure 1:**
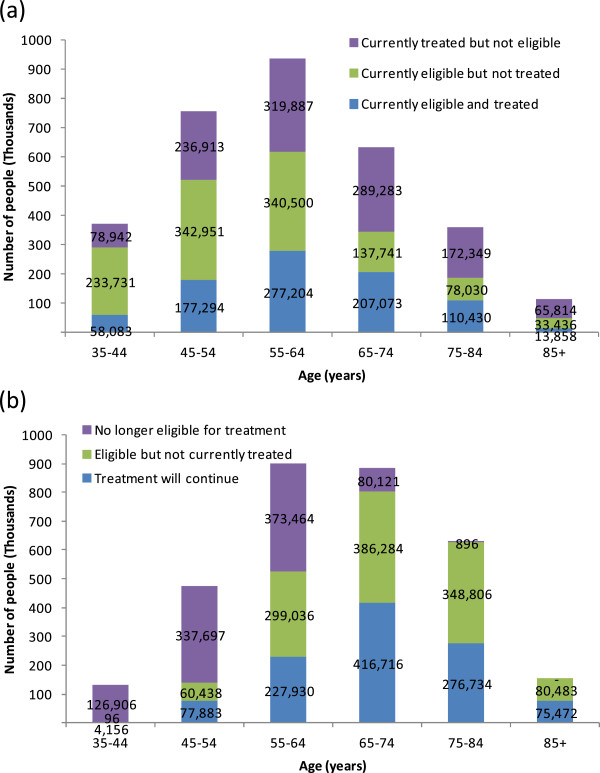
**Change in eligibility for treatment with preventive drugs.** The number of Australians already receiving treatment, newly eligible for treatment or no longer eligible for treatment, based on: **(a)** the existing single risk factor-based guidelines; and **(b)** the proposed absolute risk-based guidelines (≥5% cardiovascular disease risk).

Current cardiovascular disease prevention does not follow the existing mix of guidelines; 1.2 million Australians are missing out on treatment for which they are currently eligible and another 1.2 million are receiving treatment even though they are not currently eligible (Figure 1a). Most of those missing out on treatment under current guidelines are less than 65 years of age. A change to cardiovascular disease prevention based on absolute risk, however, would lead to an increase in average age of those eligible for treatment. If cardiovascular disease prevention is recommended for everyone with at least 5% absolute risk (Figure 1b) 2.3 million Australians will be eligible for treatment, of whom 1.1 million are already receiving cardiovascular disease drugs. However, 1 million Australians currently taking preventive drugs, mostly aged under 65 years, will no longer be eligible for treatment.

**Figure 2:**
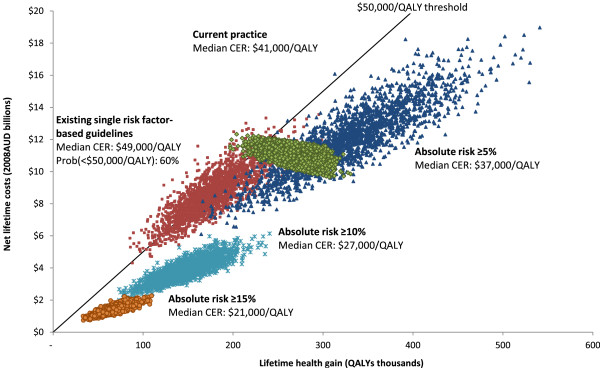
**Cost-effectiveness of cardiovascular disease prevention.** Graph shows cost-effectiveness of current practice, cost-effectiveness of existing single risk factor-based guidelines, and cost-effectiveness of prevention targeted at ≥15%, ≥10% and ≥5% absolute risk groups (NB. The scatter of points for each intervention reflects the uncertainty in the cost-effectiveness result. All points that fall under the threshold line, which is illustrated here at $50,000/QALY, are considered ‘cost-effective’).

A change in guidelines will not lead to an improvement in population health overall, but current levels of population health could be achieved at a much lower cost. Cardiovascular disease prevention based on absolute risk is more cost-effective than prevention under the current guidelines, which are based on single risk factor thresholds, and more cost-effective than the current practice (Figure 2). Recommending blood pressure-lowering drugs to everyone with at least 5% absolute risk and statin drugs to everyone with at least 10% absolute risk, can achieve current levels of population health, while saving $5.4 billion for the Government over the lifetime of the population (Table 4). If Australia could match the cheaper price of statins in New Zealand, however, Government cost savings could be as high as $7.1 billion over the lifetime of the population ($93 million in the first year).

**Table 4:** Lifetime costs, health gain and cost-effectiveness of cardiovascular disease prevention in Australia

	**Lifetime health gain (QALYs)**	**Lifetime intervention costs to Government ($billion)**	**Lifetime intervention costs to Patients ($billion)**	**Lifetime treatment costs averted ($billion)**	Cost-effectiveness* **($/QALY)**
Current practice	270,000 (220,000 to 310,000)	$12 ($12 to $12)	$2.6 ($2.6 to $2.6)	-$3.4 (−$4.4 to -$2.4)	$41,000 ($34,000 to $52,000)
Existing single risk factor-based guidelines	180,000 (120,000 to 240,000)	$7.4 ($5.1 to $9.9)	$3.5 ($2.4 to $4.7)	-$2.3 (−$3.4 to -$1.3)	$49,000 ($40,000 to $60,000)
Absolute risk (≥15%)	67,000 (44,000 to 91,000)	$1.3 ($0.9 to $1.8)	$0.8 ($0.5 to $1.0)	-$0.7 (−$1.0 to -$0.4)	$21,000 ($17,000 to $26,000)
Absolute risk (≥10%)	150,000 (97,000 to 200,000)	$3.5 ($2.4 to $4.6)	$1.9 ($1.3 to $2.6)	-$1.5 (−$2.3 to -$0.9)	$27,000 ($22,000 to $32,000)
Absolute risk (≥5%)					
– including statins <10%	330,000 (220,000 to 450,000)	$10.0 ($7.0 to $14.0)	$5.5 ($3.8 to $7.4)	-$3.7 (−$5.7 to -$2.2)	$37,000 ($31,000 to $44,000)
– excluding statins <10%	290,000 (190,000 to 390,000)	$6.5 ($4.5 to $8.6)	$4.3 ($2.9 to $5.7)	-$3.2 (−$4.8 to -$1.9)	$27,000 ($21,000 to $33,000)
Absolute risk (≥5%) assuming the cheaper price of statins in New Zealand					
– including statins <10%	330,000 (220,000 to 450,000)	$5.1 ($3.5 to $6.8)	$3.7 ($2.6 to $5.0)	-$3.7 (−$5.7 to -$2.2)	$16,000 ($12,000 to $20,000)
– excluding statins <10%	290,000 (190,000 to 390,000)	$4.7 ($3.3 to $6.3)	$3.6 ($2.5 to $4.8)	-$3.2 (−$4.8 to -$1.9)	$18,000 ($14,000 to $24,000)

NB. All values are rounded to two significant figures. Health gains and costs are presented as mean and 95% uncertainty interval, and cost-effectiveness ratio as median and 95% uncertainty interval. Costs are presented in 2008 Australian dollars. QALY – quality-adjusted life year*. A value of $50,000/QALY is often considered a threshold for cost-effectiveness in Australia.

Additional analysis with separate measures of drug efficacy for men and women did not significantly alter cost-effectiveness (Additional File 2).

## Discussion

Changing from single risk factor thresholds as a basis for primary prevention of cardiovascular disease to an absolute risk approach is highly recommended. There is potential for substantial reductions in health sector spending by more efficiently directing preventive drug therapies to those at greater overall risk. A combination of blood pressure-lowering drugs for everyone with at least 5% probability of a cardiovascular event in the next five years, could save $5.4 billion in health sector costs over the current practice, without compromising population health.

We find that prevention with blood pressure and (low-price) statin drugs is cost-effective at lower thresholds (5% five-year risk or approximately 10% ten-year risk) than are typically recommended for starting drug treatment in people without existing cardiovascular disease (e.g. 15% five-year risk in New Zealand [19] or 20% ten-year risk in the UK [52,53]). Similarly, in Argentina a combination of statin, diuretic, ACE inihibitor and aspirin was found to be cost-effective down to at least a 5% ten-year risk of an event [54] (approximately 10% five-year risk), and in the United States [55], a combination of statin, ACE inhibitor, beta-blocker and diuretic when given to men from age 55, was found to be cost-saving at all levels of risk. Thresholds for treatment, however, will differ between countries and change over time, due to variations in disease rates, in management of risk factors such as smoking, and in the factors that influence drug prices (e.g. patents) and available health care budget.

It is important to keep in mind that our estimates of current use of cardiovascular disease drugs and our calculations of the population proportions that will be eligible, rely on AusDiab data collected in 1999–2000. Not only is this dataset becoming increasingly dated, it is also reliant on self-reported use of cardiovascular disease drugs without specifying the specific drug(s) used. PBS data suggest an increasing trend in statin prescription, although trends in general practice (which are more likely to reflect use in *primary* prevention) are less apparent [31,32].

A range of other studies have also shown that absolute risk is a more effective and cost-effective approach to primary prevention than aiming to reduce blood pressure and cholesterol below threshold levels [14,56-58]. Our Australian results concur with the WHO-CHOICE findings for the WPR A region that includes Australia [14]. Taking the more recent evidence of drug efficacy into account, however, as well as the current Australian costs of drugs and costs of cardiovascular treatment, we would no longer recommend a combination of statin, diuretic, beta-blocker and aspirin. The beta-blocker would be better replaced by a calcium channel blocker and/or ACE inhibitor, both of which have greater population heath benefits for a similar level of cost-effectiveness, and recent evidence has cast doubt on the benefits of aspirin in primary prevention of cardiovascular disease [59]. New analysis of long-term outcomes from aspirin prevention trials, however, has found additional benefits of aspirin in cancer prevention [60], and further modelling work is now needed to determine if the health benefits of aspirin will outweigh the bleeding harms, at a population level, and how these new benefits will influence its cost-effectiveness if added to the package of drugs for primary prevention.

The role of statins in primary prevention of cardiovascular disease in Australia is uncertain; statins have a clear health benefit (for people at more than 10% absolute risk [61]), but they are currently an expensive addition to the prevention package in Australia. The National Health Amendment (Pharmaceutical Benefits Scheme) Bill 2010 passed by the Senate in November 2010 will guarantee a 16% price reduction for the two most expensive statins (atorvastatin and rosuvastatin) when they come off patent around 2012, but this effect is relatively small considering that Australia currently pays around five times the average price paid for statins in other OECD countries [62]. There is potential for the Government to save as much as $7.1 billion over the lifetime of the population, if Australia could match the much cheaper price of statins in New Zealand.

## Conclusion

Changing to an absolute risk-based approach to cardiovascular disease prevention in Australia, in line with other countries such as New Zealand and the United Kingdom, can save money for the Australian Government. A pure absolute risk-based approach will not lead to substantial changes in the number of Australians eligible for treatment, and the net difference in population health effects between old and new approaches will not be substantial, but it could free health dollars that could be better spent on achieving other health benefits for the population.

## Competing interests

The authors declare that they have no competing interests.

## Authors’ contributions

RC and TV conceived the cost-effectiveness analysis methods. TV designed the epidemiological model. AM and LC derived epidemiological data for model and intervention data for analysis. LC completed the modelling and data analysis, and drafted the manuscript. All authors contributed to interpretation of the data, and read and approved the final manuscript.

## Pre-publication history

The pre-publication history for this paper can be accessed here:

http://www.biomedcentral.com/1471-2458/12/398/prepub

## Supplementary Material

Additional file 1 Cost-effectiveness model and data inputs. Click here for file

Additional file 2 Cost-effectiveness results with separate effects for men and women. Click here for file
