# Supplementary material for: Improving the cost-effectiveness of cardiovascular disease prevention in Australia: a modelling study
Source: BMC Public Health. 2012 Jun 1;12:398. doi: 10.1186/1471-2458-12-398 (PMC3560211; doi:10.1186/1471-2458-12-398)
Supplement: Additional file 1 — Cost-effectiveness model and data inputs. [file 1471-2458-12-398-S1.pdf]

Additional file 1

Cost-effectiveness model and data inputs

Contents

1. Model structure 1

2. Transition probabilities 1

    2.1 Ischaemic heart disease 1

    2.2 Stroke 2

    2.3 Background mortality 2

    2.4 Disease trends 2

3. Quality of life adjustment 2

4. Costs of disease treatment 3

5. Uncertainty analysis 4

References 5

1. Model structure

The ischaemic heart disease (IHD) and stroke prevention model is a deterministic Markov model [1] that provides information on health and health care costs over the lifetime of the Australian population (or population sub-groups). The model includes four mutually exclusive health states, with transition between states based on age, sex and multivariate risk-adjusted transition probabilities (Figure 1).

Each five-year age and sex cohort initially begins in the *Alive* state before any incident IHD event (hospitalised angina or acute myocardial infarction) or stroke event (cerebral infarction or intracerebral haemorrhage). Over time, proportions of the cohort may move into an *Alive* state following an IHD event or an *Alive* state following a stroke event, with incident events divided into those that are fatal within the first year and those that survive the first year. Survivors of a first-ever event are at increased risk of death from IHD or Stroke, but may also die from other causes. Transitions are simulated annually until all of the cohort population are dead or have reached 100 years of age.

Although we do not explicitly model ischaemic and haemorrhagic sub-types of stroke as different states, differential rates of incidence and case fatality for either type of stroke event that are fatal within the first year, are taken into account in the transition probabilities.

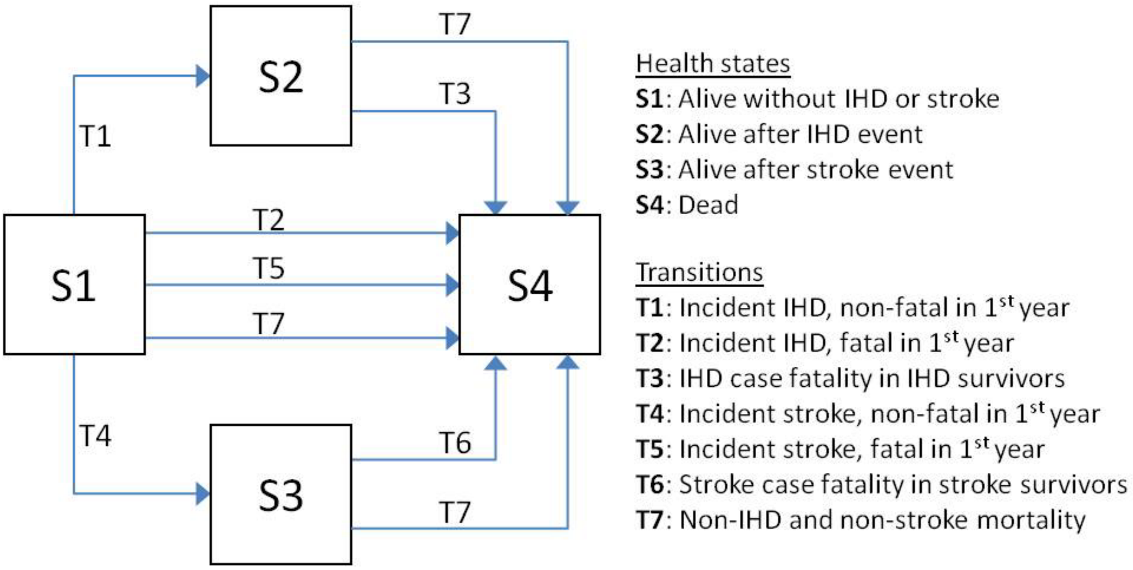

Figure 1 Markov model for simulating IHD and stroke events

2. Transition probabilities

2.1 Ischaemic heart disease

The incidence of 28-day survivors of first ever IHD is derived from recorded cases of acute myocardial infarction and unstable angina in the National Hospital Morbidity Database [2]. We then determine the incidence rate in those without previous IHD or stroke, using relative risks of incident IHD in

those with stroke form the Busselton study [3, 4] and prevalence of stroke in those without previous IHD from AusDiab [5].

Differential rates of case fatality from AMI and angina in the first 28 days are taken into account using in-hospital 28-day case fatality from the National Hospital Morbidity Database [2] and estimates of pre-hospital case fatality from the Perth MONICA study [6]. We calculate case fatality from IHD as the excess mortality in prevalent IHD survivors compared to total population mortality risks using the Western Australia Linked Hospital Database [7, 8].

2.2 Stroke

The incidence of 28-day survivors of first-ever stroke is derived from the population-based NEMESIS study [9] and Australian hospital separations data [2]. NEMESIS study incidence is scaled to a national level incidence using the ratio of hospital admissions in the NEMESIS study postcode areas at the time of the NEMESIS study, to hospital admissions Australia-wide in 2003. We adjust the stroke incidence rate to reflect the incidence rate in those without either previous IHD or stroke, using relative risks of incident stroke in those with IHD form the Busselton study [3, 4] and prevalence of IHD in those without previous stroke from AusDiab [5].

We derive separate rates of case fatality from ischaemic and haemorrhagic stroke events within the first 28 days from the NEMESIS study [9] and case fatality from stroke in 28-day survivors using the Western Australia Linked Hospital Database [8, 10].

2.3 Background mortality

Mortality from all causes other than IHD and stroke is calculated from the total number of deaths recorded by the Australian Bureau of Statistics [11] excluding deaths attributed to IHD or Stroke in the 2003 Australian Burden of Disease study [7], projected to 2008 using projections from Australian mortality data [12].

2.4 Disease trends

Future trends in IHD and stroke incidence and case fatality are based on projections made by Begg et al. [12] from observed Australian mortality between 1979 and 2003. We assume that 58% of the trend is attributable to declining incidence (i.e. due to changes in disease risk factors) and 42% to declining case fatality (i.e. due to changes in survival of those with disease) [13]. The trends are applied for the first 15 years with rates assumed to remain constant thereafter.

3. Quality of life adjustment

At each cycle of the Markov model, we calculate years of life lived within each health state, adjusting for time spent in ill health due to IHD, stroke and all other causes, to determine health gain in quality-adjusted life years (QALYs). We correct utility weights for angina, congestive heart failure, and myocardial infarction, from the Beaver dam study [14], using US population norms for background quality of life [15] (Table 1). For stroke, we correct utility weights derived in the NEMESIS

stroke study for background quality of life using AQoL population norms [16]. Average population utility weights are derived from AQoL measures for the Australian population [17] (Figure 2).

Table 1 QALY utility weights and DALY disability weights for modelled heart diseases and ulcer/GI bleed (NB. 0 represents the worst possible health state and 1 represents normal health).

Disease	QALY utility weight
Angina	0.904 [14, 15]
Congestive heart failure	0.863 [14, 15]
Myocardial infarction	0.877 [14, 15]
Stroke	0.76 [16]

NB. Beaver dam study utility weights (Quality of Well-being index) corrected for 'background disability' using US population norms (Quality of Well-being index) [15], combined multiplicatively, assuming the 'average' age for each condition is the same as in Australia [7]

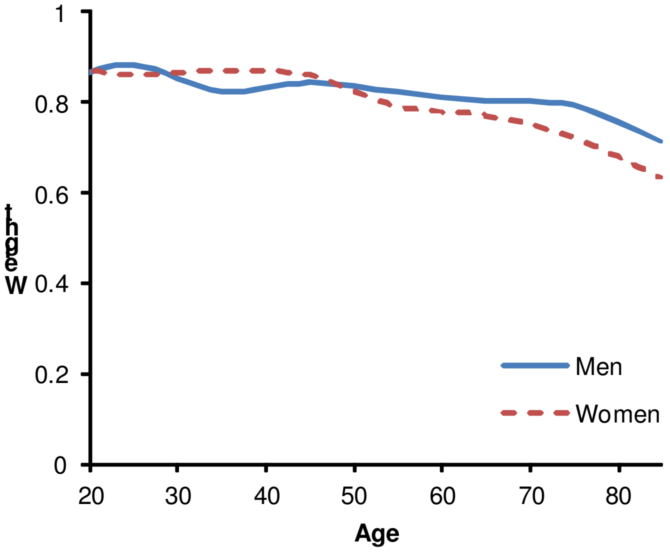

Figure 2 Average quality of life for Australians derived using the Assessment of Quality of Life (AQoL) tool [17] (NB. 0 represents the worst possible health state and 1 represents normal health).

4. Costs of disease treatment

Costs of treating IHD are determined separately for the first and subsequent years of treatment. First year costs are derived from a Victorian Government study [18] of hospital inpatient costs for IHD treatment and rehabilitation admissions, and from a study by Lim [19] of government and patient out-of-hospital costs for consultations, drugs and diagnostic procedures, and Medicare rebates for private-sector consultations and procedures. Three-quarters of first-year costs are assumed to recur in subsequent years. We derive the direct costs of treating stroke in the first year after an event and annual costs of treatment in subsequent years from the NEMESIS study of stroke costs [20]. We adjust all costs to Australian dollars in the year 2008 using health system deflators [21].

5. Uncertainty analysis

Ninety-five percent uncertainty intervals are derived for all cost and health outcome measures by Monte Carlo analysis (2000 iterations) using the Excel Add-In program @Risk (Palisade, Version 4.5). Table 2 shows the uncertainty distributions, sources and assumptions for the model input parameters.

Table 2 Model input parameters and their uncertainty distributions.

Parameter	Value<br>Mean (SE)	Uncertainty distribution	Sources and assumptions
RR of IHD with treatment			
– Statin	0.70 (0.05)	Normal (lnRR)	Meta-analyses of primary prevention trials [22-24]
– Diuretic	0.86 (0.06)		
– Calcium channel blocker	0.85 (0.04)		
– ACE inhibitor	0.83 (0.03)		
– Beta-blocker	0.78 (0.03)		
RR of stroke with treatment			
– Statin	0.81 (0.06)	Normal (lnRR)	Meta-analyses of primary prevention trials [22-24]
– Diuretic	0.62 (0.05)		
– Calcium channel blocker	0.66 (0.04)		
– ACE inhibitor	0.78 (0.07)		
– Beta-blocker	0.70 (0.04)		
First year discontinuation of drug therapy	40% (8%)	Beta	Estimate from Australian data on discontinuation of statin and blood pressure-lowering drugs [25, 26]. Standard error assumed to be 20% of point estimate.
RR IHD in stroke			
- Men	2.64 (0.07)	Normal (lnRR)	Busselton study [3, 4]
- Women	2.85 (0.04)		
Proportion of GPs participating in primary prevention program	65% (6.5%)	Beta	Estimate from GP and GP practice participation in the Practice Incentives Program scheme [27].
IHD			
- First year	$12,921	Uniform	Lim [19]. Uniform distribution assumed to vary by ±25% around mean.
- Subsequent years	$4,539		
Stroke			
- First year	$23,581	Uniform	Lim [19]. Uniform distribution assumed to vary by ±25% around mean.
- Subsequent years	$3,201		
Long GP visit [28]	$63.75	–	One visit in Year 1.
Short GP visit [28]	$33.55	–	Two visits in Year 1 (with BP-lowering drugs) and two visits in Year 2+.
Blood test (MBS Item 66512) [28]	$17.80	–	One test in Year 1 (with lipid-lowering drugs) or three tests in Year 1 (with BP-lowering drugs), and two tests in Year 2+.
Annual cost of low-dose diuretic therapy	$69.35	–	Average annual cost for the standard daily dose [29] of hydrochlorothiazide, chlorthalidone and indapamide, weighted by scripts provided in 2008 [30].
Annual cost of beta-blocker therapy	$105.85	–	Average annual cost for the standard daily dose [29] of atenolol, metropolol, propranolol, oxprenolol and pindolol, weighted by scripts provided in 2008 [30].

Annual cost of calcium channel blocker therapy	$219.00	–	Average annual cost for the standard daily dose [29] of verapimil, amlodopine (maleate), nifedipine, felodipine, amlodopine (besylate) and lercanidipine, weighted by scripts provided in 2008 [30].
Annual cost of ACE inhibitor therapy	$211.70	–	Average annual cost for the standard daily dose [29] of captopril, fosinopril, enalopril, ramipril, quinapril, lisinopril, trandolopril and perindopril, weighted by scripts provided in 2008 [30].
Annual cost of aspirin therapy	$40.15	–	Average annual cost, assuming 50% of aspirin purchased by prescription and 50% over-the-counter [30-32].
Annual cost of statin therapy	$686.20	–	Average annual cost for the standard daily dose [29] of fluvastatin, simvastatin, atorvastatin, pravastatin and rosuvastatin, weighted by scripts provided in 2008 [30].
Annual cost of statin therapy in New Zealand	$18.25	–	Average annual cost of simvastatin (40mg/day) in New Zealand [33].
Annual cost of current practice lipid-lowering therapy	$682.55	–	Average annual cost from actual PBS expenditure on lipid-lowering drugs in 2008 [30].
Annual cost of current practice blood pressure-lowering therapy	$686.20	–	Average annual cost from actual PBS expenditure on blood pressure-lowering drugs in 2008 [30]. Mix of diuretics, beta-blockers, calcium channel blockers and ACE inhibitors based on BEACH general practice data [34].

NB. All costs adjusted to 2008 Australian dollars using Australian health price deflators [21], consumer price index [35] and/or purchasing power parities [36] where relevant.

References

1. Sonnenberg F, Beck J: **Markov Models in Medical Decision Making: A Practical Guide** *Med Decis Making* 1993, **13:**322-338.
2. AIHW: **National hospital morbidity database** In *Book National hospital morbidity database* (Editor ed.^eds.). City: Australian Institute of Health and Welfare.
3. Knuiman MW, Vu HT, Bartholomew HC: **Multivariate risk estimation for coronary heart disease: the Busselton Health Study.** *Aust N Z J Public Health* 1998, **22:**747-753.
4. Knuiman MW, Vu HTV: **Risk factors for stroke mortality in men and women: the Busselton Study.** *Eur J Cardiovasc Prev Rehabil* 1996, **3:**447-452.
5. Dunstan D, Zimmet P, Welborn T, Sicree R, Armstrong T, Atkins R, Cameron A, Shaw J, Chadban S: **Diabesity and associated disorders in Australia - 2000, The Australian Diabetes, Obesity and Lifestyle Study (AusDiab).** In *Book Diabesity and associated disorders in Australia - 2000, The Australian Diabetes, Obesity and Lifestyle Study (AusDiab)* (Editor ed.^eds.). City: International Diabetes Institute; 2001.
6. McElduff P, Dobson A, Jamrozik K, Hobbs M: **The WHO MONICA Study, Australia, 1984-93: A summary of the Newcastle and Perth MONICA projects.** In *Book The WHO MONICA Study, Australia, 1984-93: A summary of the Newcastle and Perth MONICA projects* (Editor ed.^eds.). City: Australian Institute of Health and Welfare; 2000.

7. Begg S, Vos T, Barker B, Stanley L, Lopez A: **Burden of disease and injury in Australia in the new millennium: measuring health loss from diseases, injuries and risk factors.** *Med J Aust* 2008, **188:**36-40.
8. Department of Health: **Western Australian Data Linkage.** In *Book Western Australian Data Linkage* (Editor ed.^eds.). City: Department of Health (Western Australia), The University of Western Australia, Curtin University of Technolgoy, Telethon Institute of Child Health Research.
9. Thrift A, Dewey H, Macdonell R, McNeil J, Donnan G: **Stroke Incidence on the east coast of Australia : The North East Melbourne Stroke Incidence Study (NEMESIS).** *Stroke* 2000, **31:**2087-2092.
10. Katzenellenbogen JM, Vos T, Somerford P, Begg S, Semmens JB, Codde JP: **Excess Mortality Rates for Estimating the Non-Fatal Burden of Stroke in Western Australia: A Data Linkage Study.** *Cerebrovasc Dis* 2010, **30:**57-64.
11. ABS: **Deaths, Australia, 2008.** In *Book Deaths, Australia, 2008* (Editor ed.^eds.). City: Australian Bureau of Statistics; 2009.
12. Begg S, Vos T, Goss J, Mann N: **An alternative approach to projecting health expenditure in Australia.** *Aust Health Rev* 2008, **32:**148-155.
13. Unal B, Critchley JA, Capewell S: **Explaining the Decline in Coronary Heart Disease Mortality in England and Wales Between 1981 and 2000.** *Circulation* 2004, **109:**1101-1107.
14. Fryback DG, Dasbach EJ, Klein R, Klein BEK, Dorn N, Peterson K, Martin PA: **The Beaver Dam Health outcomes study: Initial catalog of health state quality factors.** *Med Decis Making* 1993, **13:**89-102.
15. Hanmer J, Lawrence WF, Anderson JP, Kaplan RM, Fryback DG: **Report of nationally representative values for the noninstitutionalized US adult population for 7 health-related quality-of-life scores.** *Med Decis Making* 2006, **26:**391-400.
16. Cadilhac DA, Dewey HM, Vos T, Carter R, Thrift AG: **The health loss from ischemic stroke and intracerebral hemorrhage: evidence from the North East Melbourne Stroke Incidence Study (NEMESIS).** *Health Qual Life Outcomes* 2010, **8**.
17. Hawthorne G, Osborne R: **Population norms and meaningful differences for the Assessment of Quality of Life (AQoL) measure.** *Aust N Z J Public Health* 2005, **29:**136-142.
18. KPMG Health Education and Community Services Group: **Cost weight study.** In *Book Cost weight study* (Editor ed.^eds.). City: Department of Human Services; 2002.
19. Lim S: **Priority setting for the primary prevention of coronary heart disease and stroke in Australia.** *PhD thesis.* Monash University, Department of Epidemiology & Preventive Medicine; 2005.
20. Dewey HM, Thrift AG, Mihalopoulos C, Carter R, Macdonell RAL, McNeil JJ, Donnan GA: **Cost of stroke in Australia from a societal perspective: results from the North East Melbourne Stroke Incidence Study (NEMESIS).** *Stroke* 2001, **32:**2409-2416.
21. AIHW: **Health expenditure Australia.** In *Book Health expenditure Australia* (Editor ed.^eds.). City: Australian Institute of Health and Welfare; 2006.
22. Brugts JJ, Yetgin T, Hoeks SE, Gotto AM, Shepherd J, Westendorp RGJ, de Craen AJM, Knopp RH, Nakamura H, Ridker P, et al: **The benefits of statins in people without established cardiovascular disease but with cardiovascular risk factors: meta-analysis of randomised controlled trials.** *BMJ* 2009, **338:**b2376-.
23. Law MR, Morris JK, Wald NJ: **Use of blood pressure lowering drugs in the prevention of cardiovascular disease: meta-analysis of 147 randomised trials in the context of expectations from prospective epidemiological studies.** *Br Med J* 2009, **338:**b1665.

24. Antithrombotic Trialists Collaboration: **Aspirin in the primary and secondary prevention of vascular disease: collaborative meta-analysis of individual participant data from randomised trials.** *The Lancet* 2009, **373:**1849-1860.

25. Simons L, Simons J, McManus P, Dudley J: **Discontinuation rates for use of statins are high [Letter].** *BMJ* 2000, **321:**1084.

26. Simons LA, Ortiz M, Calcino G: **Persistence with anti hypertensive medication: Australia-wide experience, 2004-2006.** *Med J Aust* 2008, **188:**224-227.

27. Department of Health and Ageing: **General Practice in Australia: 2004.** In *Book General Practice in Australia: 2004* (Editor ed.^eds.). City: Commonwealth of Australia; 2005.

28. MBS: **Medicare Benefits Schedule.** In *Book Medicare Benefits Schedule* (Editor ed.^eds.). City: Department of Health and Ageing, Commonwealth of Australia; 2008.

29. Law MR, Wald NJ, Morris JK, Jordan RE: **Value of low dose combination treatment with blood pressure lowering drugs: analysis of 354 randomised trials.** *BMJ* 2003, **326:**1427.

30. PBS: **Pharmaceutical Benefits Schedule.** In *Book Pharmaceutical Benefits Schedule* (Editor ed.^eds.). City: Department of Health and Ageing, Commonwealth of Australia; 2008.

31. MIMS Online: [www.mimsonline.com.au]

32. Pharmacy Direct: [www.pharmacydirect.com.au]

33. PHARMAC: **Pharmaceutical Schedule.** In *Book Pharmaceutical Schedule* (Editor ed.^eds.). City: Pharmaceutical Management Agency; 2010.

34. Senes S, Penm E: **Medicines for cardiovascular health: are they used appropriately?** In *Book Medicines for cardiovascular health: are they used appropriately?* (Editor ed.^eds.). City: Australian Institute of Health and Welfare; 2007.

35. ABS: **Consumer Price Index, Australia, June quarter.** In *Book Consumer Price Index, Australia, June quarter* (Editor ed.^eds.). City: Australian Bureau of Statistics; 2006.

36. OECD: **Purchasing Power Parities (PPPs) for OECD Countries 1980-2006.** In *Book Purchasing Power Parities (PPPs) for OECD Countries 1980-2006* (Editor ed.^eds.). City: Organisation for Economic Co-operation and Development; 2006.
