# Supplementary material for: Improving the cost-effectiveness of cardiovascular disease prevention in Australia: a modelling study
Source: BMC Public Health. 2012 Jun 1;12:398. doi: 10.1186/1471-2458-12-398 (PMC3560211; doi:10.1186/1471-2458-12-398)
Supplement: Additional file 2 — Cost-effectiveness results with separate effects for men and women. [file 1471-2458-12-398-S2.pdf]

Additional file 2

Cost-effectiveness results with separate effects for men and women

Table 1. Lifetime costs, health gain and cost-effectiveness of cardiovascular disease prevention in Australia.

	Lifetime health gain (QALYs)	Lifetime intervention costs to Government ($billion)	Lifetime intervention costs to Patients ($billion)	Lifetime treatment costs averted ($billion)	Cost-effectiveness ($/QALY)
Current practice	260,000 (200,000 to 310,000)	$12 ($12 to $12)	$2.6 ($2.6 to $2.6)	-$3.2 (-$4.4 to -$2.2)	$42,000 ($33,000 to $59,000)
Existing single risk factor-based guidelines	170,000 (100,000 to 250,000)	$7.4 ($5.2 to $9.9)	$3.5 ($2.5 to $4.6)	-$2.2 (-$3.4 to -$1.2)	$50,000 ($38,000 to $75,000)
Absolute risk (≥15%)	65,000 (44,000 to 90,000)	$1.3 ($0.9 to $1.8)	$0.7 ($0.5 to $1.0)	-$0.6 (-$1.0 to -$0.4)	$22,000 ($18,000 to $28,000)
Absolute risk (≥10%)	140,000 (96,000 to 200,000)	$3.5 ($2.5 to $4.6)	$1.9 ($1.4 to $2.5)	-$1.5 (-$2.2 to -$0.9)	$27,000 ($22,000 to $35,000)
Absolute risk (≥5%)					
– including statins <10%	320,000 (220,000 to 440,000)	$10.0 ($7.2 to $14.0)	$5.5 ($3.9 to $7.3)	-$3.6 (-$5.4 to -$2.1)	$38,000 ($32,000 to $48,000)
– excluding statins <10%	280,000 (190,000 to 390,000)	$6.5 ($4.5 to $8.6)	$4.3 ($3.0 to $5.6)	-$3.1 (-$4.7 to -$1.9)	$27,000 ($21,000 to $34,000)
Absolute risk (≥5%) assuming the cheaper price of statins in New Zealand					
– including statins <10%	320,000 (220,000 to 440,000)	$5.1 ($3.6 to $6.8)	$3.7 ($2.6 to $4.9)	-$3.6 (-$5.4 to -$2.1)	$16,000 ($12,000 to $22,000)
– excluding statins <10%	280,000 (190,000 to 390,000)	$4.7 ($3.3 to $6.2)	$3.6 ($2.6 to $4.8)	-$3.1 (-$4.7 to -$1.9)	$19,000 ($14,000 to $25,000)

NB. All values are rounded to two significant figures. Health gains and costs are presented as mean and 95% uncertainty interval, and cost-effectiveness ratio as median and 95% uncertainty interval. Costs are presented in 2008 Australian dollars. QALY – quality-adjusted life year

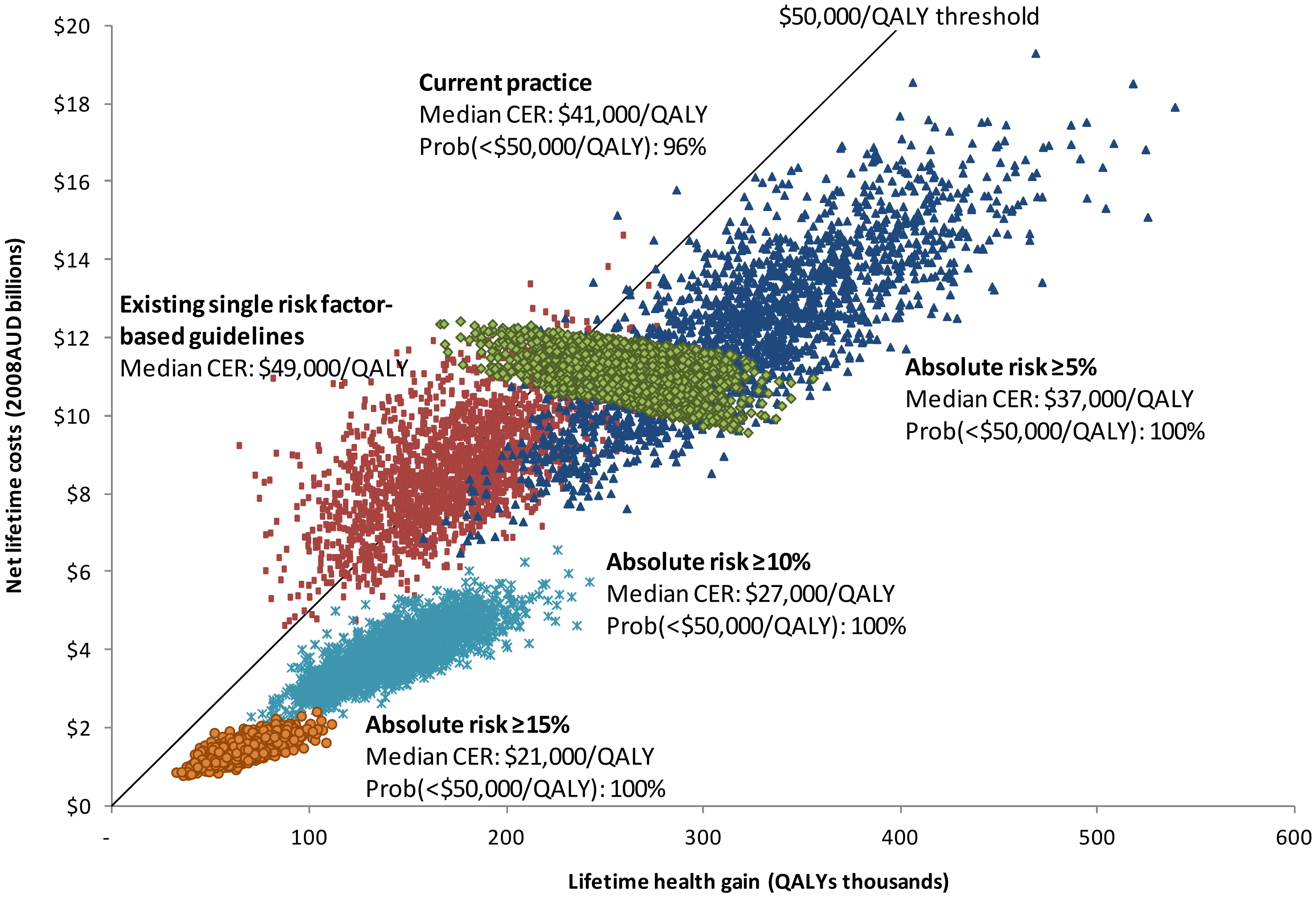

Figure 1 Cost-effectiveness of cardiovascular disease prevention
